# The German Version of the Manchester Triage System and Its Quality Criteria – First Assessment of Validity and Reliability

**DOI:** 10.1371/journal.pone.0088995

**Published:** 2014-02-24

**Authors:** Ingo Gräff, Bernd Goldschmidt, Procula Glien, Manuela Bogdanow, Rolf Fimmers, Andreas Hoeft, Se-Chan Kim, Daniel Grigutsch

**Affiliations:** 1 Clinician Scientist, Emergency Department, University Bonn, Bonn, Germany; 2 Process Management, University Bonn, Bonn, Germany; 3 Emergency Department, University Bonn, Bonn, Germany; 4 Institute for Medical Biometry, Informatics and Epidemiology, German Center for Neurodegenerative Diseases, University Bonn, Bonn, Germany; 5 Department of Anesthesiology, University Bonn, Bonn, Germany; University of Colorado Denver, United States of America

## Abstract

**Background:**

The German Version of the Manchester Triage System (MTS) has found widespread use in EDs across German-speaking Europe. Studies about the quality criteria validity and reliability of the MTS currently only exist for the English-language version. Most importantly, the content of the German version differs from the English version with respect to presentation diagrams and change indicators, which have a significant impact on the category assigned. This investigation offers a preliminary assessment in terms of validity and inter-rater reliability of the German MTS.

**Methods:**

Construct validity of assigned MTS level was assessed based on comparisons to hospitalization (general / intensive care), mortality, ED and hospital length of stay, level of prehospital care and number of invasive diagnostics. A sample of 45,469 patients was used. Inter-rater agreement between an expert and triage nurses (reliability) was calculated separately for a subset group of 167 emergency patients.

**Results:**

For general hospital admission the area under the curve (AUC) of the receiver operating characteristic was 0.749; for admission to ICU it was 0.871. An examination of MTS-level and number of deceased patients showed that the higher the priority derived from MTS, the higher the number of deaths (*p*<0.0001 / χ^2^ Test). There was a substantial difference in the 30-day survival among the 5 MTS categories (*p*<0.0001 / log-rank test).The AUC for the predict 30-day mortality was 0.613. Categories orange and red had the highest numbers of heart catheter and endoscopy. Category red and orange were mostly accompanied by an emergency physician, whereas categories blue and green were walk-in patients. Inter-rater agreement between expert triage nurses was almost perfect (**κ** = 0.954).

**Conclusion:**

The German version of the MTS is a reliable and valid instrument for a first assessment of emergency patients in the emergency department.

## Introduction

### Background

Triage in the emergency department (ED) is defined as the process of quickly categorizing patients upon arrival to determine priority for further evaluation and care. Triage Systems are either required by law or are strongly encouraged in countries with established emergency departments [1]–[4]. Most recently, there has been a trend toward intra-clinic reorganization of emergency services in hospitals across Europe. There also has been a trend toward centralization of emergency services in which previously decentralized out-patient clinics are integrated into a single interdisciplinary Emergency Department [5]. Along with this development, triage systems based on Anglo-American models are being established in German emergency rooms [6]. The Manchester Triage System has found widespread use in EDs across German-speaking Europe and has found the broadest application in German emergency rooms [7]. This system uses presentation diagrams backed by 50 algorithms, which use descriptions of complaint-complexes (such as “abdominal pain in an adult”) to target key symptoms (so-called “indicators”) and allocate the patient into one of five levels of priority. These priority levels indicate the maximal time allowed before a doctor's visit must occur [8]. The patients triaged in the highest category (red) are in need of immediate care. The next two categories (orange and yellow) have longer recommended time allowances (10 and 30 min). The two lowest categories (green and blue) have the longest recommended time allowances of 90 and 120 min from the patient's arrival to seeing a doctor. Since 2009 the MTS has been used by an automated IT solution in our ED.

### Importance

The reliable identification of critically ill patients is decisive for the quality of a triage instrument. The determined priority level should match the real need for immediate treatment. Since there is a lack of a gold standard for actual urgency, surrogate markers including rate of hospital admission or admission to ICU, mortality rate and resource use were used for examination of construct validity [6]. Recently, individual studies have tested MTS in comparison to a self-defined standard of reference (e.g., expert opinion). But even these reference standards cannot be considered to represent a gold standard, since certain evaluation criteria serving as the basis for comparison were non-standardized [9].

In addition to validity, the reliability or replicability of the results should be as high as possible so that the method of measurement can have adequate explanatory power [6]. The determination of acute need should, ideally, be the same across examiners.

### Goals of this Investigation

The Manchester Triage System was created in 1994–95 as a result of collaborative work among doctors and nurses in emergency departments from nine hospitals in Manchester, UK.

The German translation of the second edition of the Manchester Triage System has been available in book form since 2006 [8]. Since then it has found use in more than 150 German hospitals and appears to have developed into the standard system of German emergency rooms (http://www.ersteinschaetzung.de/content/verbreitung-des-manchester-triage-systems). Studies about the quality criteria validity and reliability currently only exist for the English-language version [10]–[14]. Most importantly, the content of the German version differs from the English version with respect to presentation diagrams which have been either integrated de novo or combined and changed indicators which have a significant impact on the category. Furthermore, a cultural adaption has been established. New indicators and time allowances for categories green and blue were introduced [8]. It has to be considered that a word for word translation is never possible and phrasings have to be adapted. The goal of the investigation presented here was to make a preliminary evaluation of the translated German version of the MTS in terms of its validity and inter-rater reliability.

## Materials and Methods

### Setting

The creation of a new interdisciplinary emergency department (ED) and simultaneous dissolution of multiple specialist out-patient clinics marked the beginning of optimization of emergency service structures and processes. To determine priority level the Manchester Triage System was used [8]. In cooperation with the Department of Process Management at the University Hospital Bonn the paper version of the German MTS was integrated 2009 into the hospital information system [15]. With regard to contents, there is no difference between the paper version and the automated IT solution. Furthermore the IT solution has already been certified by the German Manchester Triage Group. In total approximately 23,000 emergency patients are cared for by 14 specialties in an interdisciplinary fashion, in a space of 1300 square meters (13,993 square feet).

Gynecologic, obstetric and pediatric emergencies up to age 14 (with the exception of traumatized children and children with ENT problems) are cared for in nearby clinics and were not included.

With the introduction of the MTS, all care employees were trained in a two and one half day in-house schooling for the Manchester Triage System. New employees were given MTS training in a several-day-long program prior to working in the ED. Over the course of 24 hours nine nurses work on weekdays, eleven nurses on the weekend in a mix of specialist nurses, employees with additional qualifications and paramedics. All triage protocols of the twenty-four care employees were included in the analysis. Since 2009 the quality of triage is regularly evaluated by an audit three times a year. Since 2012, the team now has an MTS-trainer in its ranks who is responsible for the supervision of the first independent triage by MTS. The MTS-Trainer received special week-long training by the German MTS-Group that certified him to be an expert.

During the observational period from January 1, 2010 until December 31, 2011 there was no change in observational conditions, e.g. number of staff, workflow in the ED, MTS training, too, remained unchanged.

### Selection of Participants

To avoid any influence on the data, reasonable to learning effects after implementation of the MTS in 2009, triage protocols from January 1, 2010 to December 31, 2011 were analyzed. During this 24 month period a total of 45,469 patients presented at the Interdisciplinary Emergency Department and received triage with the German version of the Manchester Triage System. For evaluation of inter-rater reliability, 167 patients were evaluated over the course of two months from September to October of 2012.

### Methods of Measurement

#### Validity

All triage protocols taken from the database of the hospital information system were evaluated during the year-long observation period to analyze validity. Table 1 shows baseline characteristics. For continuous variables such as age, ED length of stay, hospital length of stay for admitted patients, the lower quartile, median, and upper quartile are given. Age was calculated based on the timestamp from the first contact minus the birth date. Length of stay in the ED was defined as the time of arrival to time of discharge or transfer is given in minutes. For patients who left the ED early without having been seen by a doctor this time-stamp was documented as discharge time. Length of hospital stay for admitted patients is given in days. The MTS priority level, sex, intervention-type, mortality rate and number of patients discharged or admitted (disposition) are given as absolute numbers and percentages. In the group of the admitted patients, two different levels of care were examined:

**Table 1:** Baseline Characteristics.

	Subcategory	N	Percentage (Frequencies)
Sex (male)		45.434	53% (24.056)
MTS Level		45.469	
	Red		1.4% (630)
	Orange		17.6% (7.999)
	Yellow		46.7% (21.221)
	Green		30.4% (13.824)
	Blue		3.9% (1.795)
Disposition		45.456	
	Discharged		67.1% (30.496)
	Admitted to normal ward		27.8% (12.650)
	Admitted to ICU / IMC		5.1% (2310)
Invasive diagnostics		45.469	
	Intra-cardiac catheter		4% (1.833)
	Endoscopy		2.3% (1.055)
Patient allocation		45.469	
	Walk-in patients		70.6% (32.105)
	Brought by paramedics		29.4% (13.364)
		**N**	**LQ / Median / UQ**
Age (Years)		45.452	27/44/64
Hospital length of stay (days)		14403	2/5/10
ED length of stay (minutes)		44.139	55/109/185

Baseline Characteristics (N =  Number of cases / LQ =  Lower Quartile / UQ =  Upper Quartile / ICU =  Intensive care unit / IMC =  Intermediate care unit).

1. Admission to intensive or intermediate care. Patients who were given CPR prior to or after arrival in the ED, as well as patients transferred directly to the operating room were defined as having been admitted to intensive care.
2. Admissions to the normal ward.

In-hospital mortality was defined as death during hospital stay.

#### Inter-rater reliability

For the assessment of inter-rater reliability cases were chosen randomly over 24 h shifts during a period of two months (September – October 2012). New admissions during MTS expert shifts were used as the basis. This selection of cases enabled the simultaneous evaluation of emergency medical history via the MTS expert (Instructor in the Competence Education Center West Germany with a special training) as well as care staff. Categorization based on MTS was conducted by each rater independently. Altogether 167 patients were observed and evaluated. The MTS categories given by the triage nurses were compared with the triage level given by the expert.

### Data Collection and Processing

All data which had a direct association with emergency care and documented in the ED were taken from the triage protocol: Baseline demographic data (age and sex), triage level, time of arrival, time of transfer (normal ward or ICU), time of discharge and patient allocation. The data base furthermore contained information on time of triage, presenting complaint, time by which the patient must be seen by the doctor, time of second survey, vital signs, pain score, and neurological / trauma triage. Data derived from hospital admission (length of hospital stay for admitted patients, death during hospital stay and number of invasive diagnostics) were also extracted from the hospital patient database. Statistical evaluation was conducted using SAS (Version 9.2; SAS Institute Inc., Cary, NC).

### Primary Data Analysis

#### Validity

Construct Validity was based upon disposition, patient mortality, length of stay (ED, hospital), number of invasive diagnostics, patient allocation and the assigned MTS level. The relationship strength between the 5 MTS levels and three possible levels of care (discharged, admitted to normal ward, admitted to ICU) was assessed using a cross-classified table, Kendall's τ -b and χ^2^ test. Likewise, evaluation of the association between MTS level and the dichotomous admission level (discharged vs. hospitalized) was conducted with the help of a cross-classified-table, Kendall's τ -b and χ^2^ test.

Level of care was considered an ordinal variable since increasing level of care reflects increasing urgency. The association of level of care and MTS was evaluated using Spearman's rank correlation coefficient. The ability of MTS to predict admission was assessed via receiver operating characteristic curves. A distinction was made here in the prediction of hospitalization in general and the predictability of admission to an ICU. The relationship between MTS level and length of stay at the ED and hospital were evaluated via Kruskal-Wallis rank sum tests.

Survival of admitted patients across the MTS categories was analyzed with the help of χ^2^ test, Kaplan-Meier estimators (product limit estimator) and the log-rank test. The duration of survival was defined as survival from the time of triage until time of death. Deceased patients in the lower MTS levels green and blue were considered separately.

Prediction of the 30-days mortality based on the 5 MTS Level was assessed by the AUC of the receiver operating characteristic.

The strength of association between the number of invasive diagnostics conducted per individual patient and the MTS score was evaluated with the χ^2^ test.

In Germany critical ill patients (e.g. severely injured patients or myocardial infarction) are cared for by prehospital emergency physicians with dedicated training and qualification (German Notarzt). In a rendezvous system, specially equipped emergency ambulances or helicopters bring the doctor to the patient. Less ill patients are transported to the ED by paramedics and ambulances. The association between the MTS categories and the level of prehospital care (Physician/ambulance, physician/helicopter, paramedic) was also assessed with χ^2^ test.

#### Reliability

Reliability is expressed in terms of inter-rater agreement. In this study we focused on consistency of ratings between triage nurses and one expert. Inter-rater agreement for ordinal scales (such as MTS) is preferably measured via weighted kappa statistic and a rank correlation coefficient. We chose Cohen's weighted κ and Spearman's rank correlation coefficient for this evaluation.

### Ethics Statement

Following consultation with the chairman of the local ethics committee (K. Racké, MD, PhD, Professor, University Bonn) the data evaluated in this study was allowed to be analyzed without approval by the ethic committee, since the analysis was purely retrospective. The General Medical Council explicitly exclude retrospective studies from approval by the ethics committee in their code of medical ethics (§15/1) (http://www.aekno.de/page.asp?pageID=57#_15).

Furthermore, by law of the German data privacy (Gesundheitsdatenschutzgesetz NW §6https://recht.nrw.de/lmi/owa/br_bes_text?anw_nr=2&gld_nr=2&ugl_nr=21260&bes_id=4283&aufgehoben=N&menu=1&sg=0#det154485), the physician may use existing patient data for retrospective analyses without explicitly asking for the consent of patients. All collected clinical data evaluated in this study were fully anonymized before analysis. Therefore, according to prior agreement with the local ethics committee and the data protection officer appointed by the University Hospital, verbal or written informed consent was not obtained.

## Results

### Baseline characteristics

No statistical differences were found among patients in relation to gender in the studies group and among groups in terms of age.

### Patient disposition and further management

**Figure 1:**
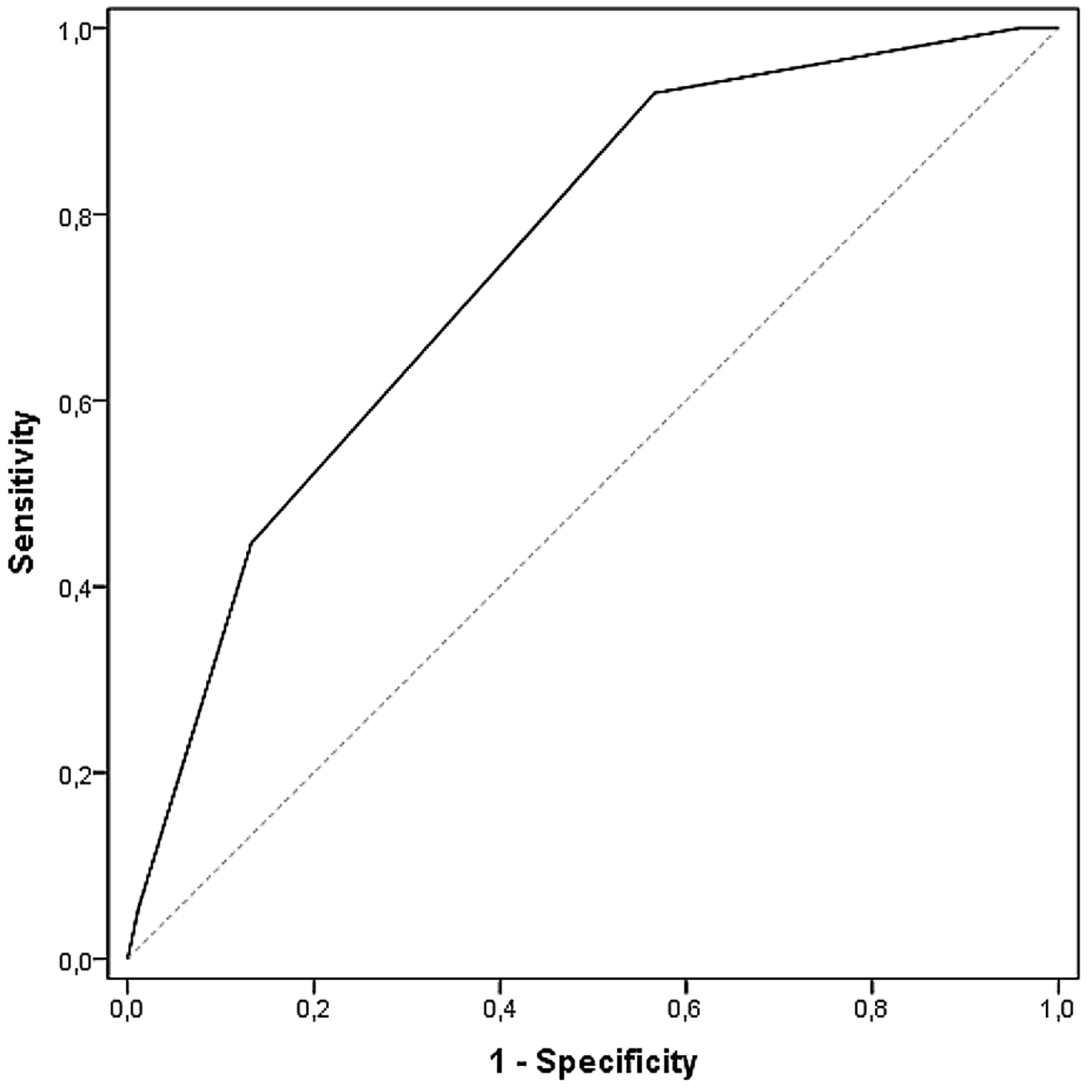
Receiver operating characteristic curves for MTS and general hospital admission. Area under the curve 0.749 (95% CI 0.744 to 0.754).

**Figure 2:**
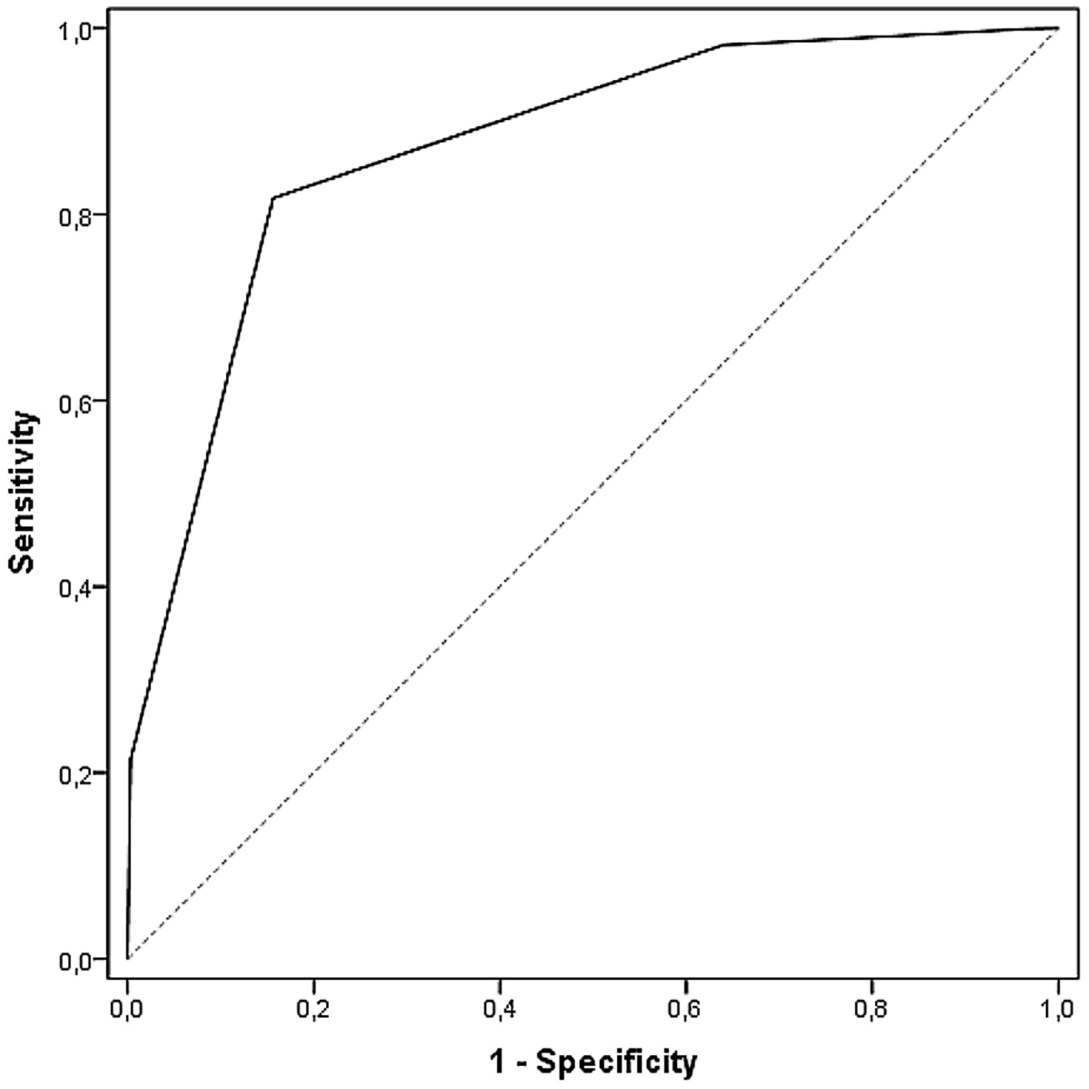
Receiver operating characteristic curves for MTS and admission to an ICU. Area under the curve 0.871 (95% CI 0.864 to 0.879).

One-hundred-percent of the patients allocated to category red were admitted to hospital, 73% of those in the orange category, 31% for those in the yellow category. In the low urgency levels blue 91% and green 87% were discharged from out-patient clinics. Patient disposition by MTS level is shown in Table 2. Application of the χ^2^ test showed that there was a clear difference between MTS level and the proportion of patients admitted into hospital in general (*p*<0.0001 / χ^2^ = 10149.27) and between hospitalization and the two levels normal ward or ICU (*p*<0.0001 / χ^2^ = 17209.23). Beyond this, the same association via Kendall's τ-b was found: Kendall's τ-b based on general admission was 0.409 (95% CI 0.401 to 0.416), focused on the two levels normal ward or ICU was τ-b = 0,423 (95% CI 0,416 to 0,431). Based on a ranking within the characteristic, an association using Spearman's correlation yielded ρ = 0.456 (95% CI 0.448 to 0.464). The receiver operating characteristic curves are depicted in Figure 1 and Figure 2. Value of MTS level for general hospital admissions is distinguished from that for ICU admissions. For hospitalization in general (Figure 1) the area under the curve was 0.749 (95% CI 0.744 to 0.754), for admission to an ICU it was 0.871 (95% CI 0.864 to 0.879).

**Table 2:** Disposition by MTS level (Continued treatment).

	MTS				
	Blue	Green	Yellow	Orange	Red
	n (%)	n (%)	n (%)	n (%)	n (%)
Out-patient	1626 (90.58)	11992 (86.83)	14746 (69.49)	2132 (26.65)	0 (0.00)
Admission to normal ward	166 (9.25)	1780 (12.89)	6095 (28.72)	4474 (55.93)	135 (21.43)
Admission to ICU	3 (0.17)	39 (0.28)	380 (1.79)	1393 (17.41)	495 (78.57)
Mortality*	5	30	154	236	193
30-Day Survival**	83.90%	92.06%	89.90%	84.50%	59.93%

*Mortality  =  Mortality admitted patients.

**Survival  =  Kaplan-Meier estimated 30-Day likelihood of survival.

**Figure 3:**
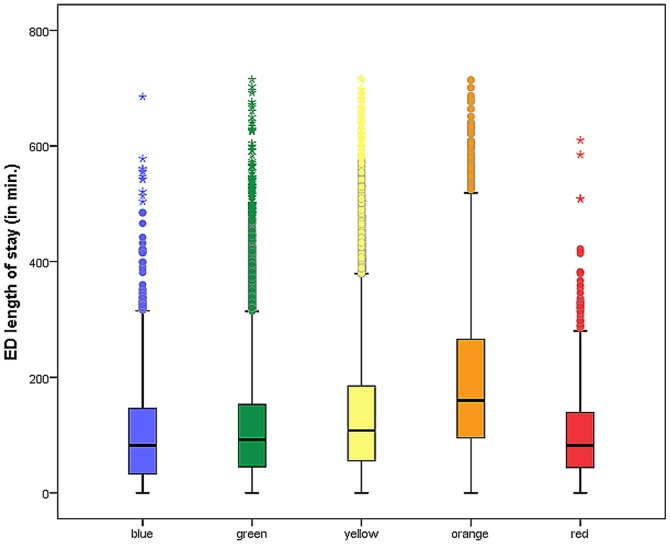
ED length of stay by MTS category. The box plots indicate the median (black line), the interquartile range (box), the smallest and largest values that are not considered outliers (whiskers, not farther away than 1.5 times the interquartile range from the first and third quartile, respectively), and values that are considered outliers (empty dots).

ED length of stay according MTS level is depicted in Figure 3. The Kruskal-Wallis rank sum test showed that ED length of stay in the individual MTS-Groups was significantly different (*p*<0.0001 / χ^2^ = 2425.06). Length of stay increased from blue to orange, and was shortest for red. Patients with MTS-Level orange stayed the longest in the ED.

**Figure 4:**
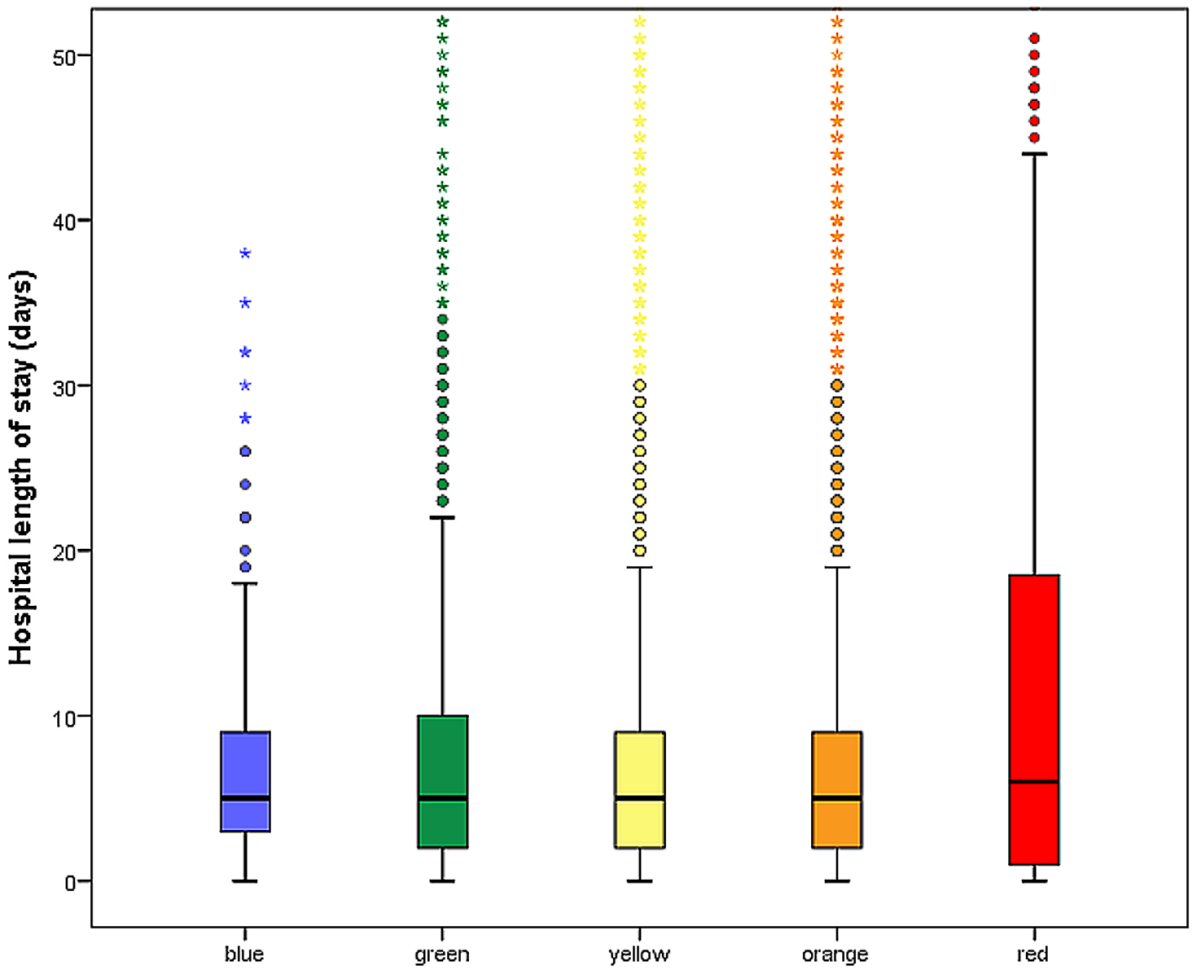
Hospital length of stay by MTS level. The box plots indicate the median (black line), the interquartile range (box), the smallest and largest values that are not considered outliers (whiskers, not farther away than 1.5 times the interquartile range from the first and third quartile, respectively), and values that are considered outliers (empty dots).

The Kruskal-Wallis rank sum test for hospital length of stay by MTS category was also significant (*p* = 0.0034 / χ^2^ = 15.75). The red patients had the longest hospital stay altogether (Figure 4).

### Mortality Risk

**Figure 5:**
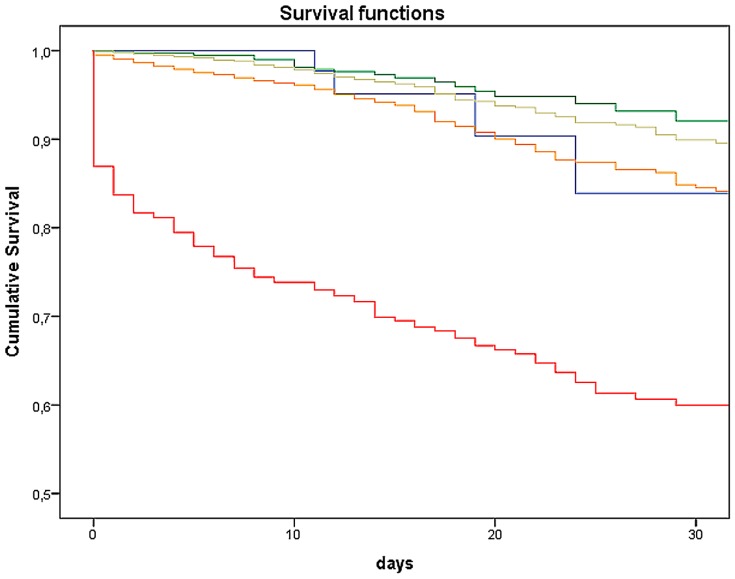
Kaplan-Meier survival curve of 30-Day likelihood of survival. Each line represents the Kaplan-Meier survival estimate for an MTS category.

**Figure 6:**
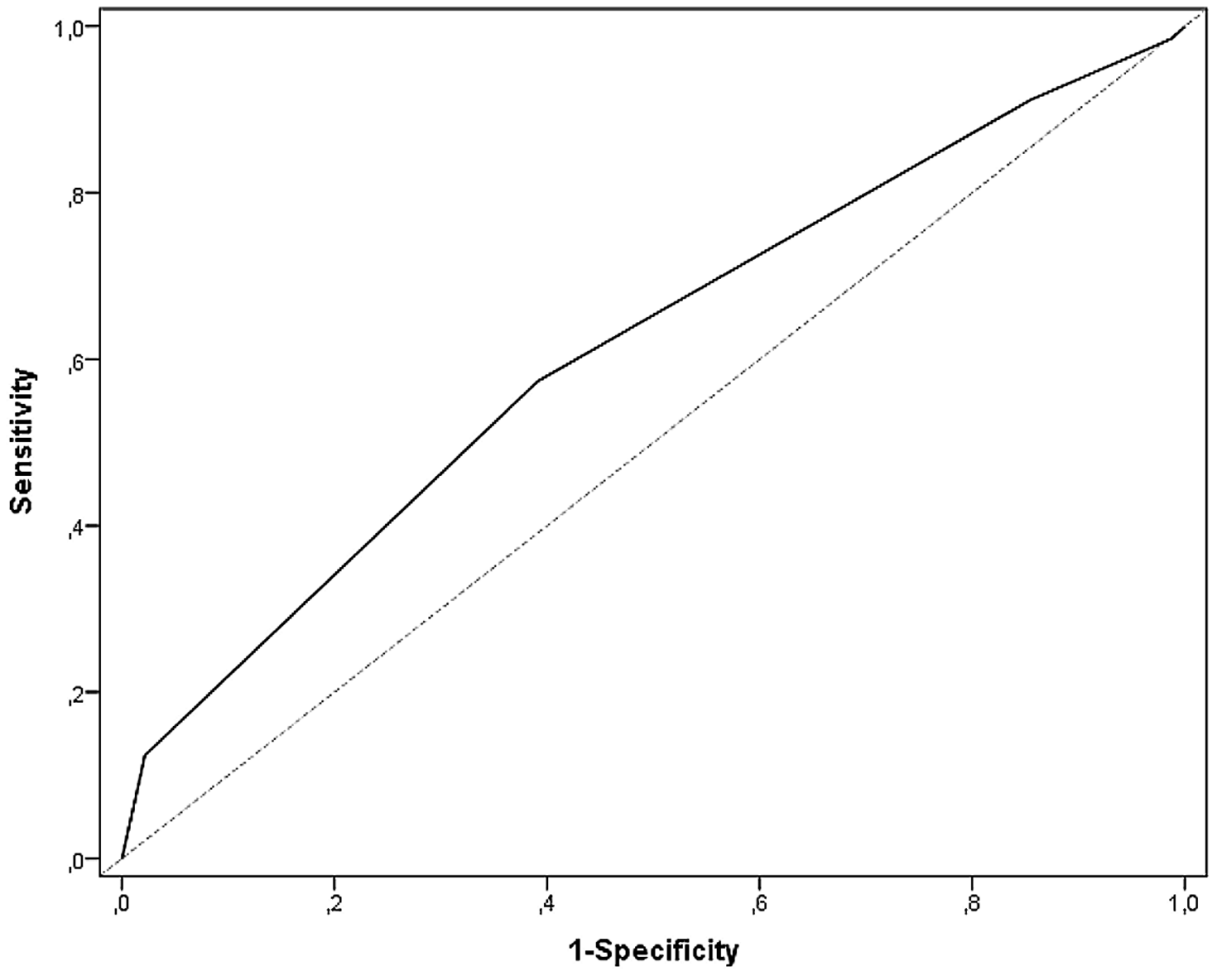
Time dependent ROC-Curve for MTS and 30-day likelihood of survival. Area under the curve 0.613.

**Figure 7:**
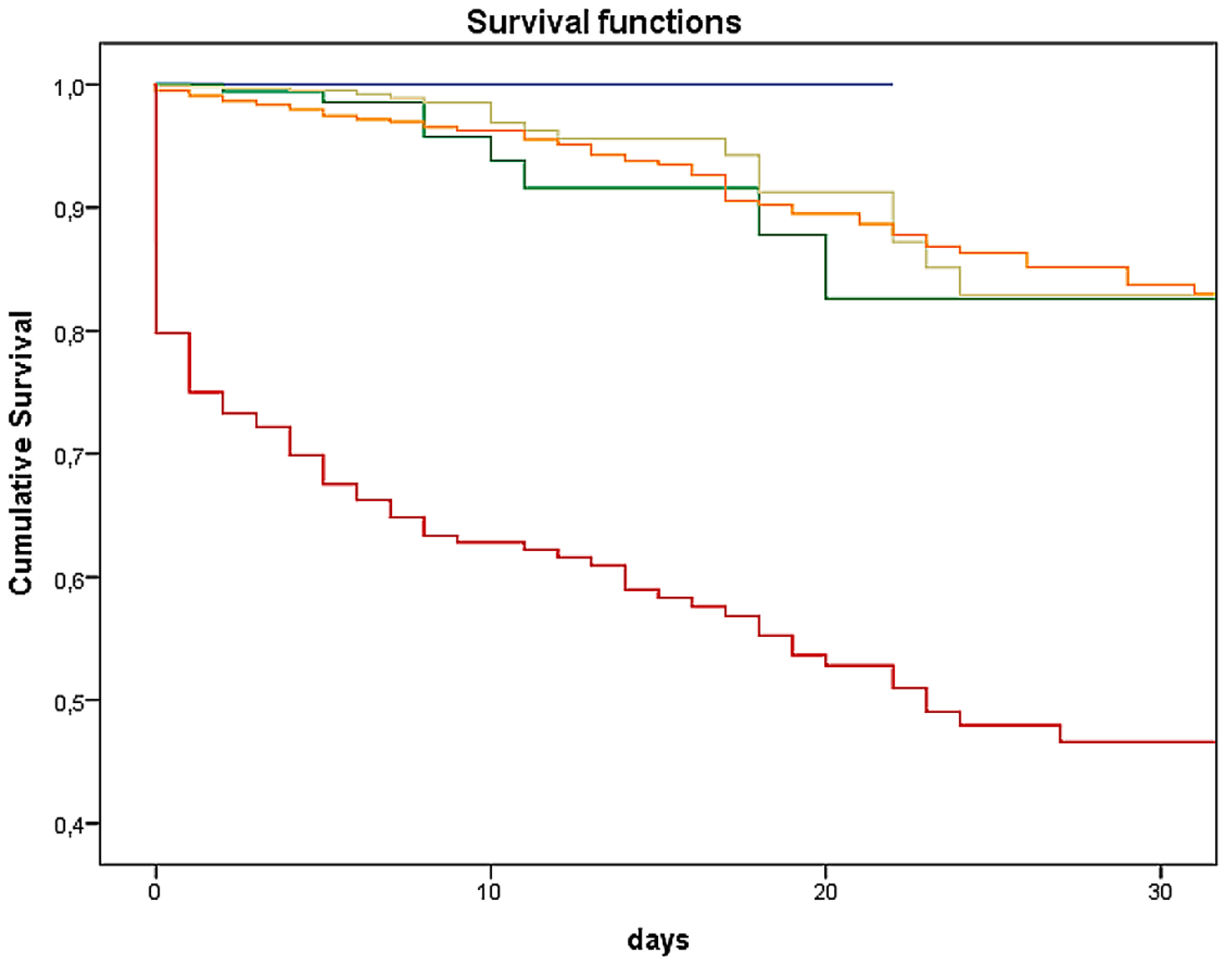
Kaplan-Meier survival curve of 30-Day likelihood of survival (subgroup cardiac patients). Each line represents the Kaplan-Meier survival estimate for an MTS category.

**Figure 8:**
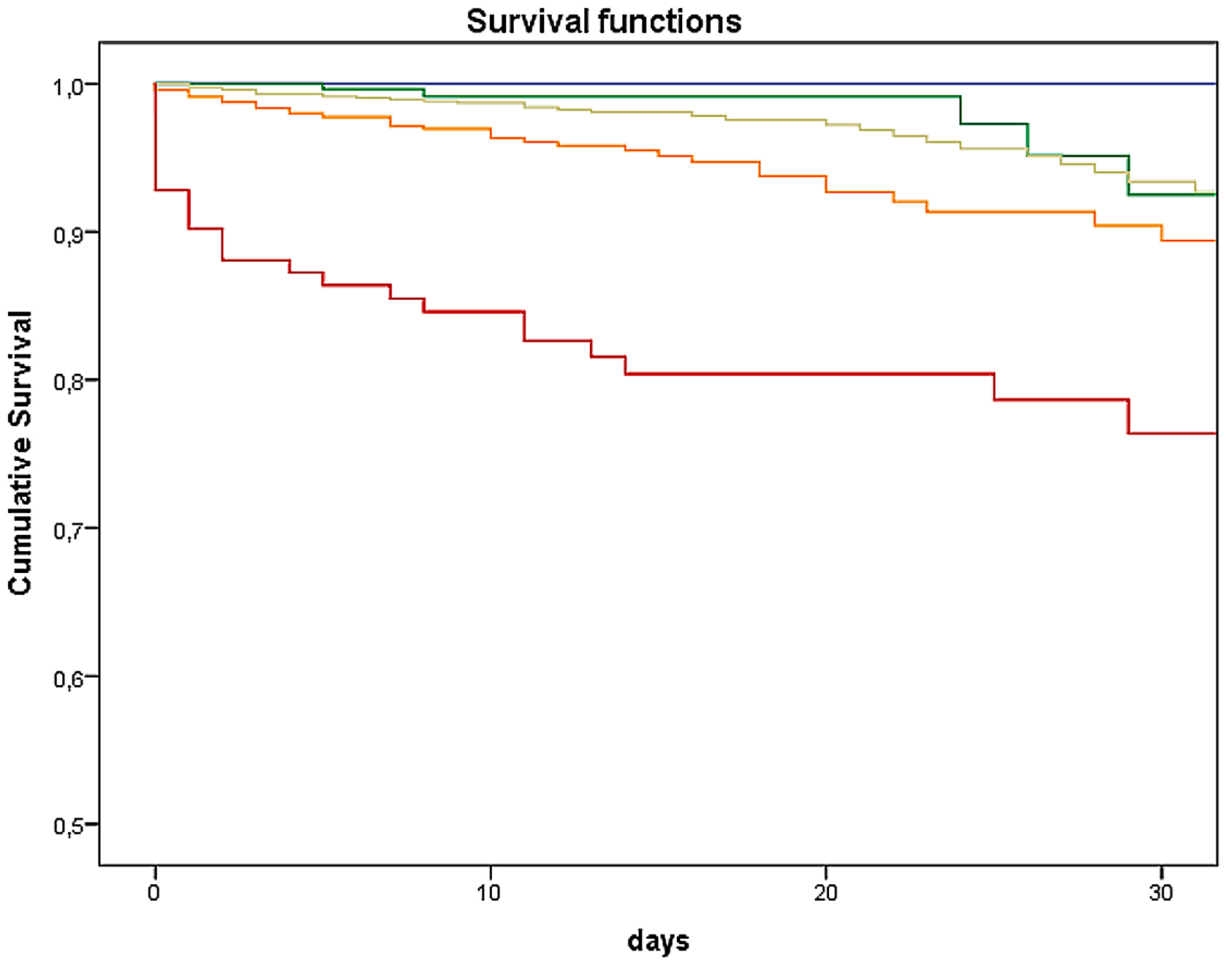
Kaplan-Meier survival curve of 30-Day likelihood of survival (subgroup surgical patients). Each line represents the Kaplan-Meier survival estimate for an MTS category.

An examination of MTS-level and number of deceased patients showed that the higher the priority derived from MTS, the higher the number of deaths (*p*<0.0001 / χ^2^ = 1157.03). Thus, within the red category, 13.8% of patients (n = 82) died within the first 24 hours. In the other categories orange till blue, together 0.22% (n = 30) died within the first 24 hours. The log-rank test showed a substantial difference in the 30-day survival among the 5 MTS categories (*p*<0.0001 / χ^2^ = 588.93). The 30-day likelihood of survival among admitted patients with lower acuity levels (MTS green and blue) was 92.06% and 83.90%, respectively. Those classified according to MTS had a likelihood of survival more than 30-days in the majority of yellow (89.90%), of orange (84.50%), and red cases (59.93%; Figure 5 and Table 2). Additionally, the time dependent ROC-Curve for MTS and 30-day likelihood of survival is shown in Figure 6. The area under the curve was 0.613. Deceased patients in the lowest urgency category showed the following distinctive characteristics: four of the blue patients died of a liver coma and one of the blue patients died of a malignant underlying disease. The situation for the group of green patients was similar, with 12 suffering from a liver coma and 18 from a malignant underlying disease. The association between duration between the time of triage and time of death in category blue showed that the first patient died after 11 days. The median of the blue group was 19.0 days after triage, the green group had a median of 11.5 days (not shown in figure). Subgroup analysis of the 30-day survival rate for those in a non-operative department (Cardiology) is depicted in Figure 7. Mortality increases with increasing level of urgency and, again, the log-rank test yielded a substantial difference in the 30-day survival among the 5 MTS categories (*p*<0.0001 / χ^2^ = 433.32). For comparison, the 30-day likelihood of survival in an operative subgroup is shown in Figure 8. Here, too, it was found that mortality increases with increasing level of urgency, and a substantial difference in the 30-day survival was determined (*p*<0.0001 / χ^2^ = 76.77).

### Invasive Diagnostics

The numbers of invasive diagnostics, intra-cardiac catheter and endoscopy used across MTS levels are indicated in Table 3. MTS category was substantial associated with intra-cardiac catheter, showed by the χ^2^ test (*p*<0.0001 / χ^2^ = 4884.70). The association between MTS category and endoscopy was also substantial (*p*<0.0001 / χ^2^ = 366.34). Categories orange and red had the highest numbers of intra-cardiac catheter and endoscopies.

**Table 3:** Numbers of invasive diagnostics (Heart Catheter / Endoscopy).

	Heart Catheter		Endoscopy	
MTS	No	Yes	No	Yes
	n (%)	n (%)	n (%)	n (%)
Blue	1789 (99.67)	6 (0.33)	1778 (99.05)	17 (0.95)
Green	13777 (99.66)	47 (0.34)	13658 (98.80)	166 (1.20)
Yellow	20928 (98.62)	293 (1.38)	20753 (97.79)	468 (2.21)
Orange	6658 (83.24)	1341 (16.76)	7653 (95.67)	346 (4.33)
Red	484 (76.83)	146 (23.17)	572 (90.79)	58 (9.21)

### Patient Allocation

**Figure 9:**
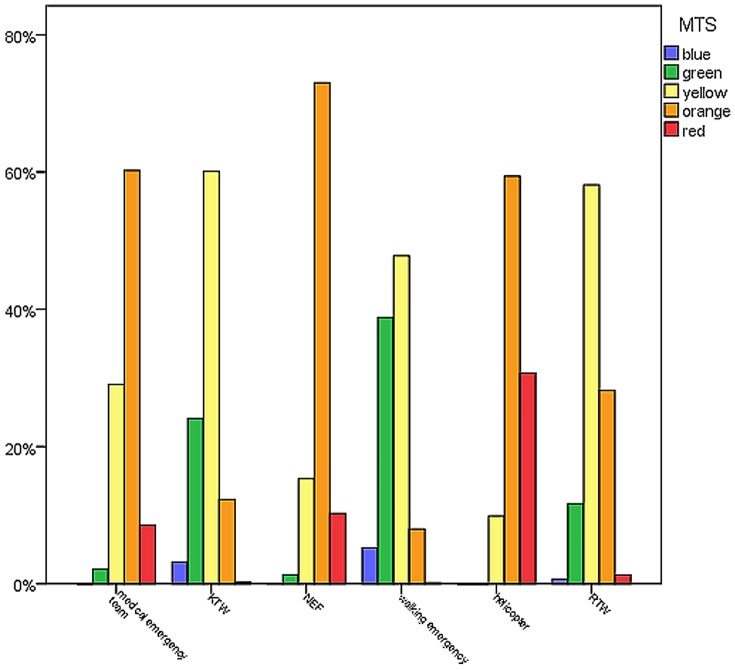
Patient allocation by MTS level. Helicopter and NEF are specially equipped emergency vehicles accompanied by an emergency physician (German Notarzt). Less ill patients are brought to the ED by paramedics and ambulances (German KTW / RTW). Category red and orange were mostly associated with prehospital contact to a doctor.

Patient allocation by MTS level is depicted in Figure 9. Association of MTS categories with level of prehospital care determined by the χ^2^ test was significant (*p*<0.0001 / χ^2^ = 17926.64). Category red and orange were mostly associated with being accompanied by an emergency physician, whereas categories blue and green were brought to the ED by an ambulance without any prehospital contact to a doctor or were walk-in patients.

### Reliability

In 97.01% of the cases, the allocation of MTS level based on expert judgment agreed with the triage level judged by the care staff. Therefore, inter-rater agreement between the expert and triage nurses was high, using both Cohen's weighted **κ** (**κ** = 0.954; 95% CI 0.912 to 0.996) and Spearman's rank correlation coefficient (p = 0,956; 95% CI 0.910 to 1.0). The inter-rater agreement between the MTS expert and the triage nurses is shown in Table 4.

**Table 4:** Inter-rater agreement between expert and triage nurses.

Weighted Kappa-Coefficient		Spearman Correlation Coefficient	
Weighted Kappa	0.9542	Correlation (r)	0.9563
95% Lower CI	0.9125	95% Lower CI	0.9101
95% Upper CI	0.9960	95% Upper CI	1.0000

### Limits

Since this study was conducted at a facility with comprehensive medical care, a high percentage of critically ill patients were treated. In particular, there were higher-than-average numbers of patients treated who had severe liver diseases. Thus, there might have been a selection bias in low-priority MTS categories blue and green.

Other potentially important factors such as age or socio-economic conditions are not part of the MTS calculation and may have an additional influence on indication for admission. Since the size of the χ^2^ - test statistic is so large, these effects may be considered statistically irrelevant in the opinions of the authors.

The doctors in charge at the Emergency Department were responsible for selecting the level of care for patients (ICU vs. normal ward) or for their discharge home, as may be the case. Since the doctors were aware of patients' triage level during emergency care, it cannot be excluded that this may have influenced the choice of further care structures via the MTS-Category. Among the higher levels of urgency, this will have only had a minor influence, since the frequency of the type of injury (e.g. polytrauma or ST-segment elevation myocardial infarction), may, make intensive care monitoring compulsory. An immediate transferal to the ICU based only on the triage-level can be excluded, since all patients undergo their primary survey in the Emergency Department. For the lower triage-categories such as “green” and “blue”, an effect is theoretically possible. In future, further, prospective blinded studies will be required to ensure the exclusion of this bias.

For patients who left the ED early without having been seen by a doctor this time-stamp was documented as discharge time. These patients can no longer be filtered out retrospectively. Since the percentage of such patients was only around 0.52% this did not confound the association between triage level and duration of stay in the ED.

## Discussion

The findings of our study are that the MTS classifications reasonable correlate with the different classes of admission. Martins et al found an increased rate of hospital admissions with increased urgency in the MTS (English version) [14]. Although Martins et al clustered the five urgency levels into two groups (red and orange  =  high priority / yellow, green and blue  =  lower priority) these data are comparable and show the same relationship. The higher the MTS level the less often patients are discharged from the ED rather than transferred to another ward [14]. Although the percentage of patients admitted per triage category of the MTS was slightly higher in our study than that reported by van der Wulp et al, our study showed the same effect.

The same high associations apply to ED length of stay, which increased across categories from blue to orange, yet was shortest for red. The authors are not aware of any comparative values from other studies using the original English version. It is logical that the red patients had the shortest duration of stay in the ED, due to their prompt transfer to emergency surgery, emergency intervention or to the ICU, as indicated. In agreement with the guidelines, patients with suspected acute coronary syndrome are assigned to MTS-Level orange based on the indicator thoracic pain. The longest duration of stay in the emergency department in the category orange can be explained by the fact that coronary patients made up a high proportion of those classified into the orange group. A second laboratory value has to be obtained to exclude heart attack in these patients, which automatically lengthened the duration of stay [15]. The association by MTS category and hospital length of stay was also significant in general, with red showing the longest hospital stay.

Mortality in our study showed increasing death rate with increasing level of MTS priority, as was shown in Martins et al [14]. Compared to the other MTS levels significantly fewer patients in the lower urgency levels died: 1.7% (*n* = 30) of the green and 2.7% (*n* = 5) of the blue patients. In the depiction of the 30-day likelihood of survival for the entire sample of admitted patients, the red patients are clearly distinct from the other categories. This observation has also been made elsewhere [14]. The cross-regional Trauma Center with many polytrauma patients and a large cardiology department conducting approximately 100 CPRs annually; these patients are classified as having the highest level of urgency. On the one hand this reflects the absolute mortality in this category, and on the other, it explains the initial high mortality during the first 24 hours. At the very least, one would expect the orange category to be somewhat nearer to the red category. According to the guidelines (12-channel EKG within 10 minutes), patients with acute coronary syndrome are allocated to category orange. The mortality of this patient group has successfully been lowered over the last several years due to the implementation of modern operating procedures and special units, like the Chest Pain Unit. This is apparently reflected in our data and also in the subgroup analysis of cardiac patients.

Regarding the deceased patients in the MTS category green and blue, the authors conclude all fatal cases within the lowest urgency category were inevitable in that they were palliative conditions. The average duration from arrival of the patient in the ED to time of death was 25.0 days for the blue category and 16.8 days for the green. Given this, in the view of the authors, the allocation of groups was correct.

For the first time this study has shown the predictive value of the 30-day mortality based on the 5 MTS levels via AUC analysis, although the value of 0.613 must be interpreted carefully. On the one hand, the moment a patient is assigned to a level of “urgent” during triage, prompt contact with a doctor is established so that measures can taken to avoid further deterioration or, in extreme cases, even death. Patients that are at the end stage of a severe disease will be placed in categories green or blue, and will “only” receive palliative measures that accompany the dying process.

This means that assigning a level of urgency involves not only an observation, but also implicates immediate carrying out of measures which can have a direct influence on mortality. For this reason, MTS should not be used as a „mortality prediction instrument“. This also holds for the ROC of the different levels of care (normal ward, ICU, Out-patient), even though the AUC-Values were better (0.74 and 0.81). In general, the ROC-Analysis with the indicated AUCs seems to demonstrate that the ordinal qualitative scale with few levels, as in the MTS, are less suitable.

Up to now, an investigation of MTS level and invasive diagnostics has not been undertaken in any publication. This association was investigated in the German MTS for the first time, showing a significant association between the MTS level and intra-cardiac catheter as well as with endoscopy. Categories orange and red had the highest numbers of intra-cardiac catheter and endoscopy. In the same vein, no study has so far investigated a triage system for its association with leading rescue methods according to the authors knowledge. We found a strong association between the MTS categories and the level of emergency vehicle. Category red and orange were mostly associated with being accompanied by an emergency physician, whereas categories blue and green were transported to the ED by an ambulance without any prehospital contact to a doctor or were walk-in patients.

Many studies have found inter-rater agreement based on retrospective evaluations of patient vignettes. The authors perceived a methodological problem in studies which require the expert to rely on notes of care staff. Vignettes do not describe the verbal and non-verbal clues which can only be observed during face-to-face interviews. Furthermore information about what constitutes an expert is often missing. In this study the expert received a special certification training by the German MTS-Group ensuring his expertise. Our study is the first to compare prospective triage agreement between triage nurses and an expert, based on live cases. We found an “almost perfect” inter-rater agreement using Cohen's weighted reliability [16]. Our results are comparable with existing literature based on the English version of the MTS, showing a substantial to almost perfect weighted kappa values [17]. In addition, using the Spearman's rank correlation coefficient in an evaluation of MTS, we found that the German MTS has a very good level of inter-rater agreement.

The MTS, as a triage priority assessment tool, was shown to be a powerful tool. In terms of patient disposition, mortality, ED length of stay and hospital length of stay showed strong associations with MTS level. Strong associations were demonstrated for the first time with two new validity criteria invasive diagnostics und patient allocation. The examination of inter-rater reliability revealed almost perfect agreement between triage nurses and the expert.

## Conclusion

The German version of the MTS in its second edition is a reliable and valid instrument for a first assessment of emergency patients in the emergency department.
